# Radiation-Associated Toxicities in Obese Women with Endometrial Cancer: More Than Just BMI?

**DOI:** 10.1155/2015/483208

**Published:** 2015-06-04

**Authors:** Savita V. Dandapani, Ying Zhang, Richard Jennelle, Yvonne G. Lin

**Affiliations:** ^1^Department of Radiation Oncology, City of Hope, Duarte, CA 91010, USA; ^2^Department of Radiation Oncology, University of Southern California, Los Angeles, CA 90033, USA; ^3^Department of Gynecologic Oncology, Obstetrics-Gynecology, University of Southern California, Los Angeles, CA 90033, USA; ^4^Genentech-Roche, 1 DNA Way, MS 444B, South San Francisco, CA 94080, USA

## Abstract

*Purpose*. The study characterizes the impact of obesity on postoperative radiation-associated toxicities in women with endometrial cancer (EC).* Material and Methods*. A retrospective study identified 96 women with EC referred to a large urban institution's radiation oncology practice for postoperative whole pelvic radiotherapy (WPRT) and/or intracavitary vaginal brachytherapy (ICBT). Demographic and clinicopathologic data were obtained. Toxicities were graded according to RTOG Acute Radiation Morbidity Scoring Criteria. Follow-up period ranged from 1 month to 11 years (median 2 years). Data were analyzed by *χ* ^2^, logistic regression, and recursive partitioning analyses.* Results*. 68 EC patients who received WPRT and/or ICBT were analyzed. Median age was 52 years (29–73). The majority were Hispanic (71%). Median BMI at diagnosis was 34.5 kg/m^2^ (20.5–56.6 kg/m^2^). BMI was independently associated with radiation-related cutaneous (*p* = 0.022) and gynecologic-related (*p* = 0.027) toxicities. Younger women also reported more gynecologic-related toxicities (*p* = 0.039). Adjuvant radiation technique was associated with increased gastrointestinal- and genitourinary-related toxicities but not gynecologic-related toxicity.* Conclusions*. Increasing BMI was associated with increased frequency of gynecologic and cutaneous radiation-associated toxicities. Additional studies to critically evaluate the radiation treatment dosing and treatment fields in obese EC patients are warranted to identify strategies to mitigate the radiation-associated toxicities in these women.

## 1. Introduction

Endometrial cancer (EC) is the most common gynecologic cancer in the United States with nearly 50,000 new diagnoses estimated in 2013 [1–3]. Radiation therapy for EC is one of the fundamental adjuvant treatment modalities and typically includes personalized field design based on patient's pathological and clinical characteristics [2, 3]. Radiotherapy strategies broadly include external beam radiotherapy and intracavitary brachytherapy. Typical treatment courses may include either modality or some combination of the two. External beam radiotherapy includes either whole pelvis radiation therapy (WPRT), with or without extended field radiotherapy (EFRT) to include the para-aortic lymph node regions [2].

A sizeable body of literature supports the use of adjuvant radiotherapy in EC to achieve local control, particularly when high risk features are present: deep myometrial invasion, histologic grades 2-3, and older age. Despite certain nuances in study design, 3 pivotal trials support the use of adjuvant radiation, either WPRT, ICBT, or some combination of WPRT/ICBT [4–8]. Interpretation of data from these studies in the context of patient's clinical and pathological characteristics forms the basis for the prescribed radiation treatment plan for EC patients [4–8].

Obesity is a growing public health problem and is a well-reported risk factor for developing EC [9, 10]. Analysis of the Multiethnic Cohort Study (MEC) found that obese women (BMI, ≥ 30 kg/m^2^) had a 3.5-fold increased risk of EC and that this magnitude of risk varied by ethnicity [11, 12]. However, little is known about the relationship between increasing BMI and the toxicity of adjuvant radiation treatments.

Patient survival after treatment for early stage EC is high (~80%) [5–7]; therefore, complications associated with treatment for EC are of particular concern for survivors and their treating oncologists. While radiation-associated toxicities can be generally classified by organ system (e.g., gastrointestinal, gynecologic, and genitourinary) and by onset (e.g., acute, delayed, and late), the specific relationship between BMI and radiation-associated toxicity is poorly understood and is the focus of the current study.

## 2. Materials and Methods

After Institutional Review Board (IRB) approval, all patients with EC treated by the radiation oncology service at our institution from 1999 to 2010 were identified for inclusion in this review. Patients were included for analysis if they met the following criteria: pathologic diagnosis of endometrial cancer, hysterectomy with bilateral salpingo-oophorectomy, receiving adjuvant radiotherapy at our institution, and having available radiotherapy records (i.e., treatment plans, dosage, and weekly symptom reports). Patients who received concurrent chemotherapy were excluded from final analysis as were patients treated with extended field radiotherapy or any patient whose radiation record was incomplete. All patients were treated with the standard pelvic 3-dimensional conformal radiation (3D-CRT) technique for WPRT incorporating the tumor bed and regional pelvic lymph nodes. No intensity modulated radiation treatment was performed for gynecologic malignancies during this time frame at the County Hospital.

Patient demographic data, including anthropometric measurements, were obtained from medical records. Radiotherapy data, including patient-reported symptoms, were culled from radiation records, and radiation-related toxicities were reviewed and graded by two radiation oncologists using the RTOG Acute Radiation Morbidity Scoring Criteria [13]. Data from weekly radiation treatment visits were evaluated, and the maximum acute radiation toxicity was scored according to the RTOG criteria. Acute side effects from radiation occur during treatment and within the first three months posttreatment. Maximum acute radiation toxicity was used as the variable to analyze because it could be assessed from patient charts during the standard weekly on treatment radiation clinic notes as well as from follow-up during the first three months after radiation treatment. There was variability in time to radiation after surgery in our patient population. There was also variability in documentation of rate of timing for onset and severity of acute side effects and so maximum acute radiation toxicity was chosen as the consistent variable. Obesity was classified using WHO criteria.

### 2.1. Statistical Analyses

Multivariate analysis using linear regression, logistic regression, and *χ* ^2^, as appropriate, was performed using JMP Pro Version 9.0.0 (SAS Institute, Cary, NC). Recursive partitioning analysis was used to model the interaction of age and BMI on acute radiation toxicities.

## 3. Results

### 3.1. Patient Characteristics

Sixty-eight evaluable EC patients referred for postoperative radiotherapy were identified for inclusion in the analysis (Table 1). The median age of the patients was 52 years (range 29–73). The majority of patients were Hispanic (71%) and had endometrioid histology (88%). 88% were overweight or obese. Patients with Stage III disease had nodal disease (50%), and 44% of the Stage III had serosal and/or adnexal involvement.

### 3.2. Radiation Therapy Characteristics

Among the sixty-eight women with EC who received postoperative radiotherapy, 26 (38%) received WPRT, 24 (33%) received ICBT, and 18 (26%) received a combination of both modalities. The median dose of whole pelvis radiation was 50.4 Gy (range 45–50.4 Gy), ICBT delivered by Low Dose Rate brachytherapy 60 Gy (range 49–88 Gy), and combined modalities 73.2 Gy (range 50.4–98 Gy).

### 3.3. BMI and Radiation-Associated Toxicities

Overall, 51 (75%) women experienced any grade radiation toxicity. The highest grade of radiation-related toxicity in our cohort was grade 2. Thirty-nine (57%) experienced grade 2 toxicities. Gastrointestinal (GI) toxicities were the most frequently reported toxicity with 35 (51%) women experiencing any grade GI toxicity. The most frequently reported symptom was diarrhea, usually requiring antidiarrheal medications (e.g., loperamide). Other toxicities included genitourinary (GU; 25, 37%), gynecologic (GYN; 13, 19%), and skin (9, 13%).

**Figure 1:**
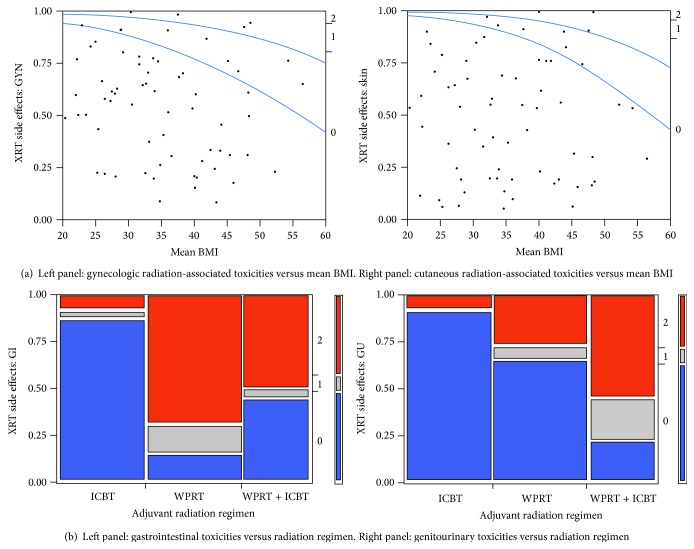
Effect of mean BMI on acute radiation toxicities. (a) Logistic regression curve modeling gynecologic and cutaneous acute toxicities versus mean BMI. (b) Bar graph depicting ratio of gastrointestinal or genitourinary acute toxicities versus adjuvant radiation treatment prescribed (WPRT, ICBT, or combination of WPRT + ICBT).

Mean BMI was associated with reported radiation-related toxicities for GYN and skin (Figure 1(a)). A higher mean BMI was significantly associated with more severe (i.e., higher grade) GYN (*p* = 0.027) and skin toxicity (*p* = 0.022). GI and GU toxicity was not associated with mean BMI on logistic regression. GI and GU toxicities were more dependent on the adjuvant radiation technique with the use of WPRT significantly associated with higher and more frequent GI (*p* < 0.0001) and GU (*p* < 0.0001) toxicities (Figure 1(b)).

**Figure 2:**
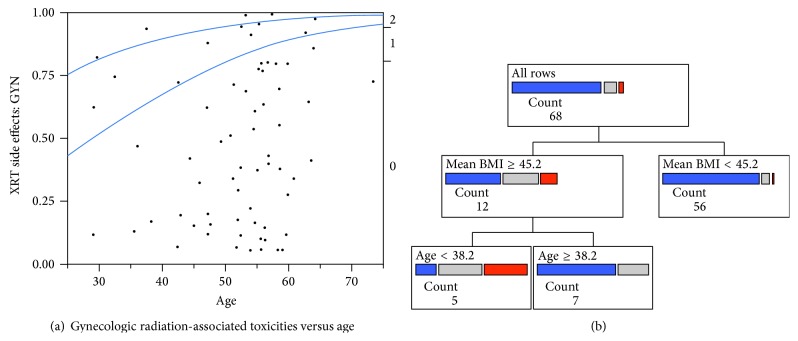
Frequency of acute gynecologic toxicities in relation to both age and mean BMI. (a) Logistic regression curve modeling acute gynecologic toxicities versus age. (b) Recursive partitioning analysis showing that gynecologic-associated toxicities increase in patients with mean BMI > 45.2 mg/m^2^ and age <38 years (blue = grade 0, grey = grade 1, and red = grade 2; count = number of patients in each subgroup).

Logistic regression also showed that GYN toxicities were significantly correlated with younger age (Figure 2(a)). There was also a relationship between younger age and increased BMI. We used recursive partitioning analysis to model the interaction of age and BMI. The first significant branch point was for BMI > 45.2 kg/m^2^, suggesting that patients above this branch point may be at particularly high risk for GYN toxicities. A second branch point was identified at age <38 years, implicating a potential age threshold at which point a treating radiation oncologist may be more attuned to early management of GYN-related symptoms (Figure 2(b)). Taken together, the highest chance of grade 2 GYN toxicity was observed in young morbidly obese women.

## 4. Discussion

The main findings from our study of this urban largely Hispanic obese population revealed that patients with increased BMI experienced more radiation-associated gynecologic and cutaneous toxicities. Recursive partitioning analysis suggests that the gynecologic toxicities may be especially increased for the morbidly obese young woman. GI and GU toxicity was associated with the use of WPRT and we did not observe any increased risk associated with increasing BMI in this cohort.

As overall survival for most patients with EC, particularly the well-differentiated Type I EC, is high (~80%) [14], the potential for long-term consequences of treatment-related toxicities is high. Whether or not obesity is an independent predictor of increased risk of recurrence or death remains controversial [12]. Therefore, given the high frequency of obesity among EC patients, a comparably high frequency of EC survivors will also be obese and likely be subject to radiation-related toxicities as well. Given the multitude of other medical comorbidities experienced and reported by obese patients, particular attention to recognize and/or prevent these complications is paramount.

Treatment field design may contribute to radiation toxicities. Jereczek-Fossa and colleagues suggested that 4-field radiotherapy may be associated with fewer late bowel toxicities; however, their findings did not retain statistical significance on multivariate analysis. Overall, 85% of the patients in their study received either WPRT + ICBT or WPRT alone and 61% had some grade 1-2 toxicity defined by RTOG criteria [15]. Our patient population reports lower (51%) than expected frequencies of radiation-associated GI toxicities among patients receiving whole pelvic radiotherapy by either 4-field box or 2-field techniques though this may be an underestimate given patient's self-reported symptoms. New technologies have emerged that may abrogate some of these toxicities. Intensity modulated radiation treatment (IMRT) can modulate where the hot spots of radiation area are placed in the treatment field and also minimize the radiation dose to nearby normal structures such as bowel [16]. None of our patients received IMRT. At our County Hospital IMRT use started in late 2009-2010 and could potentially help deliver homogeneous dose to patients with significantly high BMI in future. In lieu of IMRT for pelvic radiation, a recent paper suggests that the field-in-field (FIF) technique may also be utilized (over standard 3D-CRT used in our study) to improve dose homogeneity and reduce radiation to critical normal structures in the pelvis (i.e., bowel, bladder, and bone marrow) especially in obese patients (BMI 30–39.9). Similar to IMRT, the FIF technique could also help ameliorate acute radiation toxicities in young endometrial patients with high BMI and is now standard for other treatment sites to improve homogeneity such as breast cancer [17–19].

In addition to IMRT, image guided radiation treatment (IGRT) that can allow daily visualization of the patient anatomy and allow for tighter margins may also decrease toxicity [16]. How we use new technology in the obese population is still under investigation. Some have reported more setup errors (e.g., positioning either rotational or translational) in the obese population and a suggested planning treatment margin of 7–10 mm may miss tumor in the moderately and severely obese [16].

A recent SEER analysis shows progressive decline in the use of external beam radiation with a corresponding increase in the use of vaginal brachytherapy since 2000 [20]. This practice change may also help reduce GI and GU toxicities but there will still be a concern for GYN toxicity especially in the moderately and severely obese population. Moreover, the concern for additional radiation-associated toxicities in the obese population is associated with a commensurate trend for less radical surgery (TAH without pelvic lymph node dissection) and less radiation (trend toward more ICBT over WPRT) in this population [10, 21, 22]. Whether BMI negatively influences overall survival and surgical outcomes in the obese patient is still debatable. Some investigators have suggested that the less frequent use of adjuvant radiation in the obese population may have contributed to a decrease in cancer specific survival [21] but more data are needed to better define this relationship.

Although the current study is limited by the relatively small sample size and its retrospective design, the nature of this single institution radiation oncology practice also provides a homogeneous treatment pattern for these patients, thereby simplifying the interpretation of maximum acute radiation toxicities relative to radiation fields. All radiation-associated acute toxicities were assessed and scored by 2 radiation oncologists.

Another strength of our study is in the unique ethnic distribution of our patient population. Compared to historic randomized trials, our study population is primarily Hispanic (71%) versus the GOG-99 in which 83% were Caucasian [4, 7]. Our study begins to offer some insight into a diverse patient population not commonly enrolled in historic randomized studies. Our study population may be more comparable to the MEC because approximately 19% of the study population was Hispanic and 32% was Asian which is similar to our patient characteristics and is not mentioned in Phase 3 studies [11]. In the MEC, among the Hispanics, the endometrial cancer risk was the highest in those patients who had a BMI gain of ≥18.46% whereas for Japanese Americans a BMI gain of only ~5% was associated with a 2.17-fold higher endometrial cancer risk. The currently available MEC data is limited by the restricted radiation treatment and radiation-associated toxicity information available. However, a direct comparison of the side effects of adjuvant radiation treatment in this cohort as compared to our group may help illuminate the impact of BMI on radiation-associated toxicities in ethnically diverse populations.

Overall, adjuvant, postoperative radiation remains a key treatment modality for endometrial cancer. However, due to high survival rates especially in early stage EC, predicting and mitigating toxicities are important. Our results show that younger morbidly obese women are likely to have more toxicity and thus careful attention needs to be paid to this population.

## Figures and Tables

**Table 1:** Patient characteristics.

Characteristic	*N* (%)
Age	Median 52 years (range 29–73 years)
Ethnicity	
Hispanic	48 (71%)
Asian	13 (19%)
Caucasian	6 (9%)
African American	1 (1%)
Body mass index (BMI)	Median 34.5 kg/m^2^ (range 22.5–55.2 kg/m^2^)
Normal weight	8 (12%)
Overweight/obese	13 (19%)
Severely obese	23 (34%)
Morbidly obese	22 (32%)
Unknown	2 (3%)
Histology	
Endometrioid	60 (88%)
Nonendometrioid	8 (12%)
Histologic grade	
Grade 1	24 (35%)
Grade 2	24 (35%)
Grade 3	20 (29%)
FIGO stage	
I	25 (27%)
II	19 (28%)
III	18 (26.5%)
IV	6 (9%)
